# Supplementary material for: Benefits of psychosocial support for adolescent mothers on infant development and maternal mental wellbeing in Rakai and Kyotera, Uganda: Quasi-experimental study
Source: PLoS One. 2025 Dec 5;20(12):e0325463. doi: 10.1371/journal.pone.0325463 (PMC12680244; doi:10.1371/journal.pone.0325463)
Supplement: S1 Table — (DOCX) [file pone.0325463.s001.docx]

**Supplementary document**

**SI: Content provided during the intervention and its’ aims**

| **Session number** | **Approximate Corresponding age** | **Title and description of content** | **Aims of the sessions** |
| --- | --- | --- | --- |
| **During Pregnancy (third trimester)** | | |  |
| 1 | ANC- Third trimester | **Welcome**   - Introduction to the intervention goals and procedures - Setting group activities, expectations, and norms, identify support system for participants. - Assess participant knowledge about child development - Understanding maternal mental well being | - Prepare adolescent mothers for the intervention. - To begin the establishment of relationships for participants - To understand the available support system for the participants - Demonstrate a better level of knowledge about maternal well-being and child development at the end of the intervention |
| 2 | ANC-Third trimester | **How has my pregnancy affected me?**   - Use the mood scale to assess the mother’s moods - Activities which help to improve moods and thoughts during pregnancy - Activities for the mother and the baby in the womb; such as singing or talking to the baby - Create a birth plan - Discussions on Participants family planning | - Understand the connection between their thoughts and feelings and their outlook on life. - Understand how activities, alone or with others, can affect their moods - To improve mother- child relationship while pregnant - To enable mothers, understand key essentials they need during delivery. |
| 3 | ANC-Third trimester | **Me and my baby’s support group**   - Preparation for play and communication with one-month baby - Breast feeding demonstration and skills - Support system joins the Adolescent Mothers - Activities to demonstrate the role of support system in child up bringing - Explain the importance of support system’s active involvement in child upbringing - Discussions on Participants family planning - Discussion on how to support mother’s well-being/role of the support system | - Understand how their child will play and communicate from birth to one month old and how to support this development - Identify and communicate in which areas of their lives their support system can offer aid during pregnancy and after birth - Understand proper breastfeeding practices and swaddling of baby. |
| **Peer led sessions from when the baby is one month to six months** | | |  |
| 4 | 1 month after birth | **Will I ever get some sleep?**   - Understanding transition from pregnancy to having a baby - Importance **of sleep to both the baby and mother** - Identify common sleeping patterns and behaviour of infants - Demonstrate proper care methods: ways of swaddling, kangaroo care, positioning and dressing infants - Discus proper breast feeding as way of ensuring good sleep | - Understanding changes after giving birth - Understanding common sleeping patterns and behaviour of infants. - Articulate common issues in breastfeeding and possible solutions - particpants acquire knowledge on proper care methods: way to swaddling, kangaroo care, positioning and dressing infants. |
| 5 | 1-2 months after birth | Moms, How are we doing?   - Discussion and demonstration of proper stress management - Participate in activities which help to improve mood - Practical activities to play with and communicate with the baby - Understanding the baby’s needs and how to cope | - Understand the influence of helpful thoughts and thought patterns on mother-child relationship - Alter harmful thoughts into helpful thoughts and identify pleasant activities to improve mood - Learn how infants play and communicate at 2 months and how to support this development. |
| 6 | 2-3 months after birth | **Let’s create**   - How to improve a mothers’ well-being in the first 2 months after birth - Discuss how to play and communicate at 3 months - Explain importance of play in child development. - Learn age appropriate play in baby’s stage of development - Age specific toys at home | - Learn how infants move in their world, play and communicate at 3 months and how to support this development. - Create toys that are appropriate for their baby’s stage of development |
| 7 | 3-4 months after birth | - Let’s play and promote hygiene - Developmentally appropriate play activity with the infants - Building communication skills with the mothers - Discussion of personal and household hygiene for the participants - Demonstration of hygiene for the baby; bathing, baby belongings | - Understand how their infant will play and communicate at 4 months and how to support this development, including autonomous play and vocalizing/communication between mother and baby - Bathe infants using either soap and water, or alternatives when needed - Demonstrate how to properly wash hands and identify the 4 critical times to wash hands. |
| 8 | 4-5 months after birth | **Healthy baby feeding**   - Discuss complementary feeding practices - Discuss child development at 5-6 months - Discuss how infants communicate in distress | - Understand complementary feeding and identify locally available foods to incorporate into their babies’ diet. - Understand how their child will play and communicate at 5 months and how to support this development - Identify how infants communicate when they are in distress and how to soothe infants in distress |
| 9 | 5-6 months after birth | It takes a village to raise a healthy baby   - Demonstrate how child play and communicate at 6 months - Discuss how support system can aid the mother in supporting infant development at 6-month stage of infant’s life | - Understand how their child will play and communicate at 6 months and how to support this development - Discuss how support system can aid the mother in supporting infant development at 6-month stage of infant’s life - To help Participants promote good mental and physical well being |
| **Peer-led sessions from when the baby is 7 months to one year** | | |  |
| 10 | 6-8 months after birth | Babies on the move   - Demonstrate child play and communication at 10 months - Observe how your child’s sleeping patterns have changed since birth | - Understand how your child will play and communicate at 8 months and how to support this development, including beginning of locomotion (when the baby begins to crawl). - Observe how your child’s sleeping patterns have changed since birth |
| 11 | 8-10 months after Birth | My baby is growing   - Demonstrate child play at 10 months - Introduce more advanced methods of play with infants | - Understanding how your child will play and communicate with you at 10 months; how to support this development - Learn advanced methods of play with infants to further develop cognitive, problem-solving, and social-personal development |
| 12 | 10-12 months after birth | - My baby is turning one year old. Happy birthday - Review the content in all the previous modules - Create a plan with the PARTICIPANTS on how she can support the infant development after the sessions - Discuss options for young mother for life after 1 year of child-rearing: what can one accomplish while raising a child; what options are available and how peers can continue to support each other. | - The importance of retaining previously learned information on child development and rearing - Understanding how your child will play and communicate with you at 12 months; how to support this development - Empower the PARTICIPANTS to practice what she learnt during the project implementation time |
