# Supplementary material for: Benefits of psychosocial support for adolescent mothers on infant development and maternal mental wellbeing in Rakai and Kyotera, Uganda: Quasi-experimental study
Source: PLoS One. 2025 Dec 5;20(12):e0325463. doi: 10.1371/journal.pone.0325463 (PMC12680244; doi:10.1371/journal.pone.0325463)
Supplement: Attachment — Submitted filename: Response to Reviewers.pdf [file pone.0325463.s006.pdf]

BAMA
Babies and Mothers Alive

May 1st, 2024

Response to Reviewers

Dear Academic Editor,

Thank you for giving us the opportunity to submit a revised draft of my manuscript titled "***Benefits of psychosocial support for adolescent mothers on infant development and maternal mental wellbeing in Rakai and Kyotera, Uganda: quasi-experimental study***" to Plos one Journal. We appreciate the time and effort that you and other reviewers have dedicated to providing your valuable feedback on my manuscript. We are grateful for the insightful comments on my paper. We have been able to incorporate changes to reflect most of the suggestions provided. We have highlighted the changes within the manuscript. Here is a point-by-point response to the reviewers' comments and concerns.

Journal Requirements:

When submitting your revision, we need you to address these additional requirements.

1. Please ensure that your manuscript meets PLOS ONE's style requirements, including those for file naming. The PLOS ONE style templates can be found at https://journals.plos.org/plosone/s/file?id=wjVg/PLOSOne_formatting_sample_main_body.pdf and https://journals.plos.org/plosone/s/file?id=ba62/PLOSOne_formatting_sample_title_authors_affiliations.pdf

Response: Documents have been formatted using the PLOS ONE's style reequipments. See Revised Manuscript with Track Changes and Manuscript

We note that the grant information you provided in the 'Funding Information' and 'Financial Disclosure' sections do not match. When you resubmit, please ensure that you provide the correct grant numbers for the awards you received for your study in the 'Funding Information' section.

Response: This has been modified in the system,

3. Please state what role the funders took in the study. If the funders had no role, please state: ""The funders had no role in study design, data collection and analysis, decision to publish, or preparation of the manuscript.""
If this statement is not correct you must amend it as needed.
Please include this amended Role of Funder statement in your cover letter; we will change the online submission form on your behalf.

Response: Funding was provided by;
Grand Challenges Canada's Saving Brains Program. This was received by DM, https://www.grandchallenges.ca/, Grant number: SB-1810-20437

Additional funding from Brick by Brick Partners, received by MS, https://togetherwomenrise.org/programfactsheets/brick-by-brick-partners/, No grant number

Staff paid by the grants include; DM, EN, EN, MP, RK

The funders had no role in study design, data collection and analysis, decision to publish, or preparation of the manuscript.

4. In the online submission form, you indicated that [Data will be available upon formal request for the first Author through email: esther.nakyaze@gmail.com for researchers who meet the criteria for access to confidential data.].
All PLOS journals now require all data underlying the findings described in their manuscript to be freely available to other researchers, either 1. In a public repository, 2. Within the manuscript

itself, or 3. Uploaded as supplementary information.
This policy applies to all data except where public deposition would breach compliance with the protocol approved by your research ethics board. If your data cannot be made publicly available for ethical or legal reasons (e.g., public availability would compromise patient privacy), please explain your reasons on resubmission and your exemption request will be escalated for approval.

Response: Data will be uploaded as supplementary information in the system; S2 Dataset. Ages and Stages data, S3 Dataset. Self-Reported Questionnaire, S4 Dataset. Demographic data

5. Please ensure that you include a title page within your main document. You should list all authors and all affiliations as per our author instructions and clearly indicate the corresponding author.

Response: Title page included in the main document (see page 1 of the Manuscript)

6. Please include captions for your Supporting Information files at the end of your manuscript, and update any in-text citations to match accordingly. Please see our Supporting Information guidelines for more information: http://journals.plos.org/plosone/s/supporting-information.

Response: Supporting documents uploaded accordingly following the guidelines.

7. Please review your reference list to ensure that it is complete and correct. If you have cited papers that have been retracted, please include the rationale for doing so in the manuscript text, or remove these references and replace them with relevant current references. Any changes to the reference list should be mentioned in the rebuttal letter that accompanies your revised

manuscript. If you need to cite a retracted article, indicate the article's retracted status in the References list and include a citation and full reference for the retraction notice.

Response: Reference list updated

Removed reference;

Lesser J, Escoto-Lloyd S. Health-related problems in a vulnerable population: pregnant teens and adolescent mothers. *Nurs Clin North Am.* 1999;34(2):289-299.

New reference added:

Okalo, P., Arach, A. A., Apili, B., Oyat, J., Halima, N., & Kabunga, A. (2023). Predictors of Unintended Pregnancy Among Adolescent Girls During the Second Wave of COVID-19 Pandemic in Oyam District in Northern Uganda. *Open access journal of contraception*, *14*, 15–21. https://doi.org/10.2147/OAJC.S399973

Additional Editor Comments:

Dear authors,

Thank you for addressing Adolescent Mothers' Mental well-being and Infant Developmental Outcomes. I have some comments that I hope will help in improving the manuscript.

Introduction:

In the introduction section, the authors should state the rate of unplanned and unwanted pregnancies among teenagers in Uganda. Also, they should report those rates among their participants in the methods section.

Response: Rate of unplanned and unwanted pregnancies among teenagers in Uganda and rates among their participants in the methods section stated (see page 2 line 51-52).

Methods:

Further description of the SRQ-20 is needed. Please state the scale's total score, its cut-off point (if there is one), and the meaning of the total score.

Response: The SRQ -20 is described on page 10. The tool caries a total score range of 0-20. A "Yes" response to experience of a mental health symptom was transcribed to carry 1 score while "No" was transcribed caries carry 0 score. Based on the context, some studies and programs have created cut offs, however this study focused on using the tool to assess maternal mental wellbeing at different assessment points.

Results:

Two groups should have been compared in terms of the family support they had already received. This can be stated as a weak point of the study.

Response: Results on family support received during the study period is available, although not presented in this paper, this will be present in a separate paper.

The sentence in lines 192-3 is incomplete and needs revision.

Response: modifications made.

In the first paragraph, the authors state, "Our results also show statistically significant reductions in maternal symptoms of mental illness at term and 6-months postpartum." However, in Table 3, no significant reduction is found in the scores of the control group.

Response: The scores were significant, results in the table as well. See table 3 column 11, 12 and 13.

We look forward to hearing from you in due time regarding our submission and to respond to any further questions and comments you may have.

Sincerely

Esther Nakyaze
First Author
