# Supplementary material for: Benefits of psychosocial support for adolescent mothers on infant development and maternal mental wellbeing in Rakai and Kyotera, Uganda: Quasi-experimental study
Source: PLoS One. 2025 Dec 5;20(12):e0325463. doi: 10.1371/journal.pone.0325463 (PMC12680244; doi:10.1371/journal.pone.0325463)
Supplement: Attachment — Submitted filename: Response_to_Reviewers_auresp_3.pdf [file pone.0325463.s009.pdf]

BAMA
Babies and Mothers Alive

October 20th, 2024

Response to Reviewers

Dear Academic Editor,

Thank you for giving us the opportunity to submit a revised draft of my manuscript titled "***Benefits of psychosocial support for adolescent mothers on infant development and maternal mental wellbeing in Rakai and Kyotera, Uganda: quasi-experimental study***" to Plos one Journal. We appreciate the time and effort that you and other reviewers have dedicated to providing your valuable feedback on my manuscript. We are grateful for the insightful comments on my paper. We have been able to incorporate changes to reflect most of the suggestions provided. We have highlighted the changes within the manuscript. Here is a point-by-point response to the reviewers' comments and concerns.

Reviewer comments

Reviewer #3: Thank you for addressing the previous comments satisfactorily. I have minor remarks, recommended to change during publication process
Abstract- remove reference no 4 from abstract at the end of second sentence.
Response: Reference has been removed (see page one, line 26 of the tracked manuscript).

Don't start sentence with number such as 789 adolescent
Response: This has been modified (see page two, line 43 of the tracked manuscript).

Since, intervention and curriculum development is a part of the methods of the experimental study, it is highly recommended moving those sections to the methods part than keeping as introduction.
Response: Description of the study moved to the methods section as per the reviewer recommendation (See page 9-12 of the tracked manuscript).

In study diagram fig 1, loss to follow up has separated with the number and lost babies. I assume lost babies is reason of loss to follow up, not separate number as study population is mother not the babies. So it is recommended to keep total loss to follow up as total number 85 and reason as lost babies - .. , maternal death .. and other reason.
Response: This has been modified, see figure 1, page 16.

Masaka-Kyotera Road, Bulinda Village Kalisizo Town, Kyotera District
P.O Box 972, Masaka
0200902073
https://babiesandmothersalive.org/

Reviewer#2

Abstract

Line 34: 'Usual care' can be briefly described.

Response: Modification made. See page 2 of the tracked manuscript. Care as usual has been further described on page 12 and 13 of the methods section

Line 34: For the abstract to be stand-alone, even the 'psychosocial support programming' can be briefly described (what exactly did it entail?) Its possible to do this without a major increase in the word count.

Response: Modification made. See page 2 of the tracked manuscript.

Line 39: not advisable to start a sentence with a number.

Response: Modification made. See page 2 of the tracked manuscript.

Lines 40-45: it is always advisable to include the effect size; what was the score in each group? This is important in enabling the reader to interpret the findings, something which is not possible with p-value alone.

Response: These have been included, see page 2 of the tracked manuscript.

Methods

Line 105: the standard care needs to be described. What does the usual care for such mothers' entail, in the study setting? This is important in delineating the incremental value of the intervention.

Response: Standard ANC and PNC described on page 12 of the tracked manuscript.

Lines 109-117: who participated in the development of the curriculum? Was it validated? How was the validation process? Creation of curriculums have specific steps, which may be different from development of training packages, for instance.

Response: The curriculum was developed by program staff with expertise in public health, adolescent health, maternal and child health, clinical psychology, and monitoring and evaluation, with technical guidance from two consultants—a pediatrician and a psychologist. To ensure the curriculum was relevant to the Ugandan context, development sessions included contributions

from facility and community health workers, as well as mothers of adolescents in the Rakai and Kyotera Districts.

The MAP curriculum was primarily based on the World Health Organization's Care for Child Development Manual, Northwestern University's Mothers and Babies curriculum, and Uganda's Infant and Young Child Feeding Guidelines (IYCF). Additional literature on infant and maternal development was also incorporated, with all materials consolidated and adapted to fit the local context and adolescent target population.

After development, the curriculum was validated through a pilot implementation in a controlled setting to assess its effectiveness. Feedback was collected and analyzed, which informed the creation of the final version. Following validation, we implemented the curriculum and conducted bimonthly meetings with users to evaluate its effectiveness and make necessary updates.

134: A reference for the Care for Child Development Curriculum is necessary here.

Response: care of child development reference included (see page 9-10 of the manuscript).

Lines 165: Study setting: how far are the two districts from each other? Any possibility of contamination during the interventions?

Response: The districts are approximately 30kms from each other and thus minimal possibility of contamination. This information has been included under study setting.

Sample size calculation: usually in scientific publications, this section is worded differently from the study protocol. I would advise instead of showing the formula, the authors reword it as follows: *In order to have power of 80% to detect a difference in developmental milestones between the two groups of at least 10%, with an alpha level of .05, and proportion of xxx among the control of 50%, we required a minimum sample size of 389.*

The authors can improve on that.

Response: Rewording has been done, see page 8 of the tracked manuscript.

Ethical consideration: how did you deal with the issue of informed consent, since a substantial proportion of your participants were expected to be minors?

We followed the guidance form the Uganda National Council of Science and Technology (UNCST)regarding "emancipated minors." According to the UNCST, emancipated minors are individuals under the age of 18 who have been legally granted independence from their parents

or guardians. This status allows them to make decisions regarding their health, education, and other personal matters without parental consent.

- Emancipated minors can consent to medical treatment, enter contracts, and make independent decisions regarding their lives.
- The criteria for emancipation can vary, but it often requires demonstrating maturity, financial independence, and the ability to live independently.
- In the context of research, emancipated minors may have the authority to provide informed consent, impacting ethical guidelines and approval processes.

Results and discussion

The control group had a higher proportion of mothers with no children. Any impact the authors postulate this could have had on the findings of the interventions?

Response: There are likely no effects of this on the findings. We controlled any covariates during data analysis. Data were analyzed using the difference-in-difference method. We ran a regression for the entire sample and then separately for observations at 2 months, 6 months, and 12 months:

$$y_{it} = \beta_0 + \beta_1 Post_6 + \beta_2 Post_{12} + \beta_3 Treat_i + \beta_4 Post_6 \times Treat_i + \beta_5 Post_{12} \times Treat_i + e_{it}$$

Standard errors were clustered at the mother-level and maternal mental wellbeing at enrolment, towards birth and 6-months post-birth using multivariate regression.

We look forward to hearing from you in due time regarding our submission and to respond to any further questions and comments you may have.
Sincerely

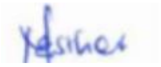

Esther Nakyaze
First Author
