# Supplementary material for: Benefits of psychosocial support for adolescent mothers on infant development and maternal mental wellbeing in Rakai and Kyotera, Uganda: Quasi-experimental study
Source: PLoS One. 2025 Dec 5;20(12):e0325463. doi: 10.1371/journal.pone.0325463 (PMC12680244; doi:10.1371/journal.pone.0325463)
Supplement: Attachment — Submitted filename: Response to Reviewers_EN_4.12. 2024.pdf [file pone.0325463.s010.pdf]

BAMA
Babies and Mothers Alive

December 4th, 2024

Response to Reviewers

Dear Academic Editor,

Thank you for giving us the opportunity to submit a revised draft of my manuscript titled "***Benefits of psychosocial support for adolescent mothers on infant development and maternal mental wellbeing in Rakai and Kyotera, Uganda: quasi-experimental study***" to Plos one Journal. We appreciate the time and effort that you and other reviewers have dedicated to providing your valuable feedback on my manuscript. We are grateful for the insightful comments on my paper. We have been able to incorporate changes to reflect most of the suggestions provided. We have highlighted the changes within the manuscript. Here is a point-by-point response to the reviewers' comments and concerns.

Academic Editor's comments

We appreciate your diligence in thoroughly addressing the reviewers' comments, and we are pleased with the revisions made so far. However, before we can proceed with accepting the manuscript for publication, we kindly request that you conduct a final, thorough proofreading of the manuscript to ensure clarity and adherence to the journal's standards.

We recommend focusing on the following:

Comment: Sentence Structure: Simplify and refine sentences for better coherence and readability.
Response: This has been Addressed

Grammatical Accuracy: Correct any typographical and grammatical errors.
Response: This has been Addressed

Punctuation: Ensure appropriate use of full stops, commas, and other punctuation marks.
Response: This has been Addressed

We look forward to hearing from you in due time regarding our submission and to respond to any further questions and comments you may have.
Sincerely

Masaka-Kyotera Road, Bulinda Village Kalisizo Town, Kyotera District
P.O Box 972, Masaka
0200902073
https://babiesandmothersalive.org/

BAMA
Babies and Mothers Alive

Esther Nakyaze
First Author

Masaka-Kyotera Road, Bulinda Village Kalisizo Town, Kyotera District
P.O Box 972, Masaka
0200902073
https://babiesandmothersalive.org/
