# Supplementary material for: Benefits of psychosocial support for adolescent mothers on infant development and maternal mental wellbeing in Rakai and Kyotera, Uganda: Quasi-experimental study
Source: PLoS One. 2025 Dec 5;20(12):e0325463. doi: 10.1371/journal.pone.0325463 (PMC12680244; doi:10.1371/journal.pone.0325463)
Supplement: Attachment — Submitted filename: Response to Reviewers_EN_29.04. 2025.pdf [file pone.0325463.s011.pdf]

BAMA
Babies and Mothers Alive

April 24th, 2025

Response to Reviewers

Dear Academic Editor,

Thank you for giving us the opportunity to submit a revised draft of my manuscript titled "***Benefits of psychosocial support for adolescent mothers on infant development and maternal mental wellbeing in Rakai and Kyotera, Uganda: quasi-experimental study***" to Plos one Journal. We appreciate the time and effort that you and other reviewers have dedicated to providing your valuable feedback on my manuscript. We are grateful for the insightful comments in my paper. We have been able to incorporate changes to reflect most of the suggestions provided. We have highlighted the changes within the manuscript. Here is a point-by-point response to the reviewers' comments and concerns.

Journal Requirements:

Please review your reference list to ensure that it is complete and correct. If you have cited papers that have been retracted, please include the rationale for doing so in the manuscript text or remove these references and replace them with relevant current references. Any changes to the reference list should be mentioned in the rebuttal letter that accompanies your revised manuscript. If you need to cite a retracted article, indicate the article's retracted status in the References list and include a citation and full reference for the retraction notice.

Response: The reference list has been reviewed and updated, no retracted articles. Four references have been updated to provide the latest updated references. (*see reference list in the tracked manuscript)*

1.

Removed reference:

Masaka-Kyotera Road, Bulinda Village Kalisizo Town,
Kyotera District
P.O Box 972, Masaka
0200902073
https://babiesandmothersalive.org/

Gideon R. Factors Associated with Adolescent Pregnancy and Fertility in Uganda: Analysis of the 2011 Demographic and Health Survey Data. *American Journal of Sociological Research 2013.* 2013;3(2):30-35.

And replaced with:

Chemutai V, Musaba MW, Amongin D, Wandabwa JN. Prevalence and factors associated with teenage pregnancy among parturients in Mbale Regional Referral Hospital: a cross-sectional study. Afr Health Sci. 2022 Jun;22(2):451-458. doi: 10.4314/has.v22i2.52. PMID: 36407378; PMCID: PMC9652643.

This is mainly to provide an updated reference

2.

Removed reference:

Nabukhonzo P. Uganda Demographic and Health Survey (UDHS). In:2012:1-10 and123.

Okalo, P., Arach, A. A., Apili, B., Oyat, J., Halima, N., & Kabunga, A. (2023).

Replaced with:

Amongin, D., Benova, L., Nakimuli, A. *et al.* Trends and determinants of adolescent childbirth in Uganda- analysis of rural and urban women using six demographic and health surveys, 1988–2016. *Reprod Health* **17**, 74 (2020). https://doi.org/10.1186/s12978-020-00925-8

This is mainly to provide an updated reference

3.

Removed reference:

Kwon MK. Parenting stress and related factors of employed and non-employed mothers with infants. *Korean Journal of Childcare and Education.* 2011;7(2):19-41

Replaced with

Flaherty SC, Sadler LS. Parenting Stress Among Adolescent Mothers: An Integrative Literature Review. *Western Journal of Nursing Research*. 2022;44(7):701-719. doi:10.1177/01939459211014241

This is mainly to provide an updated reference

4.
Reference
Removed reference:
http://www.brickbybrick.org/

Replaced with
Babies and Mothers Alive Foundation Website
https://www.babiesandmothersalive.org/

This is mainly to provide an updated reference

Additional Editor Comments:

I would like to thank the authors for making edits to the manuscript in reponse to the previous editorial and peer-reviewed comments. The study findings are very relevant and interesting. Since, I have just stepped in as the Academic Editor, I would suggest some further edits to the draft which are as follows:

Abstract:
1. Abstract in its current form is too lengthy and needs to be more succinct.

Response: This has been edited. *See page 1 and 2 of the manuscript*

2. Spell out WHO in the abstract when used first time.

Response: This has been modified- (see page 2)

3. Specify that the estimates reported for the ASQ infant development domains are Mean difference (MD) and not means. Also specify the time point (for e.g. "The difference-in-difference estimations reveal that the intervention group outperformed the control group in all five ASQ infant development domains, with statistically significant results ($p<0.01$) across 2-12 months: "

Response: This has been included

Introduction:
1. Please add a reference here: "Beyond these contributing factors, adolescent

pregnant mothers also have low antenatal care (ANC) attendance and limited knowledge of child development and care as compared to older pregnant mothers."

Response: Reference has been included (see page 4 line 80)

2. Spell out "BAMA" when used in the manuscript for the first time.
Response: This has been included (see page 4 line 98)

3. I would suggest if the authors could update the references in the introduction section to more recent ones, if available.
Response: These have been updated (all references in the entire article have been updated

Methods:
1. Under 'Study design and setting', please specify that Rakai Hospital was chosen as the intervention and Kalisizo Hospital was chosen as the control group.
Response: This has been included (see page 5 of the tracked manuscript)

Statistical analysis:
1. Does this statement "Pearson’s chi squared tests were used to compare categorical variables greater than 5 cells and Fishers exact tests for variables less five cells" mean that Pearsons chi-square test was used for variables with a cell-count of > 5 and fishers exact test was used for cell counts < 5 ? if this is the case, please make this sentence more clearer.
Response: This has been included (see page 12 of the tracked manuscript)

Results:
1. In figure 1 (study diagram), please add the numbers followed up at 2 months, 6 months and 12 months
Response: this has been included, *see page 22 of the tracked manuscript*

Discussion:
1. I would suggest that if the authors could add some discussion around the sustainability and integration of this intervention at a larger scale for longer run.

Response: this has been included, *see page 22 of the tracked manuscript*

Masaka-Kyotera Road, Bulinda Village Kalisizo Town,
Kyotera District
P.O Box 972, Masaka
0200902073
https://babiesandmothersalive.org/

2. I would suggest the authors to add some insight on how the baseline difference in number of children at enrolment, education level, religion, and family size between the intervention and control groups could impact the results, if at all, in the 'Limitations' section.

Response: Although the differences heighted were available, we used a regress equation for the entire sample and then separately for observations at 2 months, 6 months, and 12 months:

$$y_{it} = \beta_0 + \beta_1 Post_6 + \beta_2 Post_{12} + \beta_3 Treat_i + \beta_4 Post_6 \times Treat_i + \beta_5 Post_{12} \times Treat_i + e_{it}$$

The outcome (y_{it}) is the sum of the questions across each domain of communication, gross motor, fine motor, problem solving, and personal-social. $Post_6 = 1$ if the observation is in the 6th month, $Post_{12} = 1$ if the observation is in the 12th month, and $Treat_i = 1$ if the mother was in the intervention group. An F-test was conducted to test the null hypothesis of equality across groups.

This catered for all the confounders.

Response: This is described under page 13 of the tracked manuscript)

Overall, please make sure that the acronyms are spelled out once when these are used for the first time in the draft and then can be used as acronyms throughout the text. For e.g. acronyms of WHO, MAP, LMICs etc. have been spelled out multiple times throughout the manuscript.

Response: These have been spelt out across all the document

We look forward to hearing from you in due course regarding our submission and to responding to any further questions and comments you may have.

Sincerely

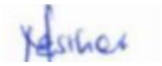

Esther Nakyaze
First Author

Masaka-Kyotera Road, Bulinda Village Kalisizo Town,
Kyotera District
P.O Box 972, Masaka
0200902073
https://babiesandmothersalive.org/

Masaka-Kyotera Road, Bulinda Village Kalisizo Town,
Kyotera District
P.O Box 972, Masaka
0200902073
https://babiesandmothersalive.org/
