# Supplementary material for: Benefits of psychosocial support for adolescent mothers on infant development and maternal mental wellbeing in Rakai and Kyotera, Uganda: Quasi-experimental study
Source: PLoS One. 2025 Dec 5;20(12):e0325463. doi: 10.1371/journal.pone.0325463 (PMC12680244; doi:10.1371/journal.pone.0325463)
Supplement: Attachment — Submitted filename: Response to Reviewers_EN_23.06.2024.docx [file pone.0325463.s008.docx]

**June 3^rd^ , 2024**

**Response to Reviewers**

Dear Academic Editor,

Thank you for giving us the opportunity to submit a revised draft of my manuscript titled “***Benefits of psychosocial support for adolescent mothers on infant development and maternal mental wellbeing in Rakai and Kyotera, Uganda: quasi-experimental study***” to Plos one Journal. We appreciate the time and effort that you and other reviewers have dedicated to providing your valuable feedback on my manuscript. We are grateful for the insightful comments on my paper. We have been able to incorporate changes to reflect most of the suggestions provided. We have highlighted the changes within the manuscript. Here is a point-by-point response to the reviewers’ comments and concerns.

Additional Editor Comments:

In the results section, two groups should have been compared in terms of the family support they had already received. It is important to report those results.

Response: the study didn’t assess family support received by the adolescent mothers before the study. This has been included as a study limitation-See page 20, line 418-420.

Note: *The study assessed family support at 6 months post-delivery using Multidimensional Scale of Perceived Social Support (MSPSS)-See page 09, line 211-215. These results have been submitted as S5_Family support during the study.*

The sentence in lines 211-13 is still incomplete and needs revision.

Response: the sentences have been modified.

We look forward to hearing from you in due time regarding our submission and to respond to any further questions and comments you may have.

Sincerely

Esther Nakyaze

First Author
