# Supplementary material for: Benefits of psychosocial support for adolescent mothers on infant development and maternal mental wellbeing in Rakai and Kyotera, Uganda: Quasi-experimental study
Source: PLoS One. 2025 Dec 5;20(12):e0325463. doi: 10.1371/journal.pone.0325463 (PMC12680244; doi:10.1371/journal.pone.0325463)
Supplement: Attachment — Submitted filename: Reviewer comments_300724.docx [file pone.0325463.s007.docx]

**Reviewer comments**

**Abstract**

Line 34: ‘Usual care’ can be briefly described.

Line 34: For the abstract to be stand-alone, even the ‘psychosocial support programming’ can be briefly described (what exactly did it entail?) Its possible to do this without a major increase in the word count.

Line 39: not advisable to start a sentence with a number.

Lines 40-45: it is always advisable to include the effect size; what was the score in each group? This is important in enabling the reader to interpret the findings, something which is not possible with p-value alone.

**Methods**

Line 105: the standard care needs to be described. What does the usual care for such mothers’ entail, in the study setting? This is important in delineating the incremental value of the intervention.

Lines 109-117: who participated in the development of the curriculum? Was it validated? How was the validation process? Creation of curriculums have specific steps, which may be different from development of training packages, for instance.

134: a reference for the Care for Child Development Curriculum is necessary here.

Lines 165: Study setting: how far are the two districts from each other? Any possibility of contamination during the interventions?

Sample size calculation: usually in scientific publications, this section is worded differently from the study protocol. I would advise instead of showing the formula, the authors reword it as follows: *In order to have power of 80% to detect a difference in developmental milestones between the two groups of at least 10%, with an alpha level of .05, and proportion of xxx among the control of 50%, we required a minimum sample size of 389.*

The authors can improve on that.

Ethical consideration: how did you deal with the issue of informed consent, since a substantial proportion of your participants were expected to be minors?

**Results and discussion**

The control group had a higher proportion of mothers with no children. Any impact the authors postulate this could have had on the findings of the interventions?
